# Virtual reality, machine learning, and statistical modeling reveal an association between multiple concussion history and impaired perceptual decision-making

**DOI:** 10.1371/journal.pone.0358042

**Published:** 2026-09-21

**Authors:** Gary B. Wilkerson, Mohammad Joghataee, Ashish Gupta

**Affiliations:** 1 Department of Health & Human Performance, University of Tennessee at Chattanooga, Chattanooga, Tennessee, United States of America; 2 Department of Business Analytics & Information Systems, Harbert College of Business, Auburn University, Auburn, Alabama, United States of America; Instituto Tecnológico y de Estudios Superiores de Monterrey: Tecnologico de Monterrey, MEXICO

## Abstract

Current clinical assessment methods are insufficiently sensitive for detection of subtle post-concussion impairments in perceptual-cognitive function. The purpose of this study was to search for any perceptual response metrics associated with concussion history that might increase understanding of altered brain-behavior relationships. Immersive virtual reality test data were aggregated for 202 healthy adolescents and young adults (116 males, 86 females) who participated in different studies over a 2-year period. Left- versus right-directed neck rotation, arm reach, and step-lunge responses to sequential presentations of 2 types of horizontally moving visual stimuli were measured in terms of time to initiation of body segment movement (perceptual latency [PL]), as well as response completion (response time [RT]). Speed-accuracy tradeoff was represented by rate correct per second for PL (RCS-PL) and RT (RCS-RT) of neck and arm movements, and across-trial inconsistency was represented by PL variability (PLV) and RT variability (RTV). Both supervised machine learning and theory-based statistical regression methods were used to identify metrics that best discriminated between participants who reported a history of no concussion (NC), single concussion (SC), NC + SC, or multiple concussions (MC). Additionally, statistical regression was used to assess a theoretical relationship between metrics believed to align with components of the drift-diffusion computational model of decision-making. The best metric for discrimination between NC + SC and MC was Neck RCS-PL. Neck PLV values demonstrated a strong inverse logarithmic correlation with Neck RCS-PL (r = –0.796, *P* < 0.001). The Neck RCS-PL and Neck PLV behavioral metrics may have relevance to the two components of the drift-diffusion computational model of perceptual decision-making, and their combination may be associated with a cumulative and persisting deficiency after having sustained more than one lifetime concussion.

## 1. Introduction

Despite accumulating evidence that mild traumatic brain injury can have adverse effects on brain function for months or years beyond the resolution of clinical symptoms, sport-related concussion is still widely viewed as a phenomenon that transiently disrupts normal brain function without causing long-term impairment. Factors contributing to the view of SRC as a benign condition include the highly subjective and transient nature of its acute effects and the inadequacy of current clinical assessments for the detection of subtle and persistent impairments that may increase susceptibility to a future injury [1,2]. A history of prior concussion is known to be a strong predictor of subsequent concussion occurrence [3], as well as musculoskeletal injury [4]. A delayed and clinically silent pathophysiological response sensitizes the immune system and increases vulnerability to a more severe subsequent concussion [5]. There is strong evidence that multiple concussions have cumulative adverse effects on cerebral blood flow [5], microstructural integrity of axons [6], hippocampal volume [7], visual attention [8], oculomotor function [9], cognitive and sensorimotor processes [5,10], motor learning [11], psycho-affective mood [12], sleep quality [13], susceptibility to lower extremity injury [14], and risk for future development of a neurodegenerative disorder [15].

Abnormalities in brain microstructure and function can be detected by advanced neuroimaging technologies and electrophysiologic testing, but their cost and limited accessibility make them impractical for risk screening and longitudinal monitoring of changes in status of individuals who do not exhibit any obvious indicators of neurological dysfunction [5,8]. Because behavioral tests currently used in the clinical setting are not sufficiently sensitive to detect subtle impairment [1,2,5,16–18], there is a critical need for a cost-effective and sensitive method that will identify individuals who possess elevated risk for cumulative brain damage and musculoskeletal injury [19–21]. Although the exact mechanism of elevated injury risk is not understood [16,17,22,23], disrupted integration of neural signals associated with perceptual, cognitive, and motor processes is believed to be the key factor [11,19,24,25].

Dual-task testing that simultaneously imposes both a cognitive demand and a motor demand is a widely used strategy to detect impaired neural processing in the clinical setting [26], which is manifested by worse performance on one or both of the component tasks in comparison to single-task performance (i.e., dual-task cost). Such tests typically involve measurement of walking gait velocity or maintenance of postural stability while engaged in a cognitive task that requires a series of verbal responses. Ideally, the cognitive and motor processes should be integrated to perform a goal-oriented task that reflects the demands of a competitive sport environment [27,28]. Furthermore, the speed of time-constrained perceptual decisions that generate accurate motor responses to visual inputs needs to be quantified [1,27,29,30].

Assessment and training of the functional capabilities of athletes have traditionally focused on improvement of physical attributes, such as muscle strength, power, endurance, and flexibility. Few studies have assessed the potential contributions of cognitive processes to prevention of injury. The combination of visual detection and cognitive interpretation of environmental stimuli is often referred to as a “perceptual-cognitive” skill, whereas the term “cognitive-motor” has been used to refer to the interrelated neural processes involved in decision-making and execution of complex movement responses. Because cognition is integral to both perception and motor programming, the term “perceptual-motor” is often used to represent the observable result of integrated stimulus-response processing. A key consideration for the design of a clinical test of perceptual-motor performance, as well as selection of the metrics used to represent performance, is a clear understanding of latent neural mechanisms that have been linked to goal-directed behaviors [20,31–33]. The “drift-diffusion model” of two-alternative forced-choice decision-making has been demonstrated to provide a strong link between neural and behavioral data [34].

Sport participation requires athletes to rapidly process visual inputs to make numerous decisions based on limited information [35], and under highly demanding temporospatial constraints that often present an elevated level of injury risk [30]. Although little is currently known about how decision-making and sensorimotor control interact [36], immersive virtual reality (VR) offers a means to quantify the speed, accuracy, and consistency of sequential behavioral responses to moving visual stimuli that each present a two-alternative forced-choice decision [37]. The inherent tradeoff between decision speed and accuracy appears to be an important aspect of behavioral performance that reflects latent neural processes [38], and inconsistency of decision times across trials (i.e., intra-individual variability) has been associated with microstructural axon damage [39], as well as potential vulnerability to further injury after concussion [40]. A key advantage of immersive VR performance metrics may be their derivation from the integrated delivery of perceptual, cognitive, and motor demands, compared to those derived from dual-task test procedures that impose distinctly separate tasks simultaneously [41,42].

Numerous factors impose major limitations on research efforts to improve concussion diagnosis, prognosis, and long-term clinical management [43]. Concussion has historically been associated with a discrete injury event, typically coinciding with a head impact, but delayed emergence of subtle and persistent abnormalities in brain function greatly complicates diagnostic accuracy [44]. Almost all information available to clinicians for concussion diagnosis comes from patient-reported symptoms, which athletes are frequently reluctant to disclose [45], and existing clinical assessment metrics have questionable value for detection of subtle impairments [1,2]. Even with a sufficiently sensitive clinical measure, the acquisition of test data from enough concussed individuals to minimize the probability of a Type II hypothesis test error presents a formidable challenge [46]. Furthermore, a reductionist and hypothesis-driven approach is unlikely to identify complex, multivariable, and non-linear relationships that may be necessary for advancement of concussion management [47].

Supervised machine learning (ML) may identify complex interrelationships among disparate factors affecting brain processing efficiency, which are less likely to be revealed through reductionist research methods that are more focused on isolating the hypothesized effects of an independent variable [48]. However, data inputs to supervised ML (i.e., features) generate output that does not necessarily incorporate domain knowledge [49], which has been described as an uninterpretable “black box” process [50]. A theory-based analysis of the same dataset was independently conducted by one of the authors, which used well-understood statistical methods to identify key metrics in the context of domain knowledge [50]. Thus, the purpose of this exploratory and data-driven study was to use both supervised ML and theory-based statistical regression methods to identify any immersive VR performance metrics associated with self-reported concussion history, as well as any relationships among them, that might increase understanding of the mechanism responsible for elevation of post-concussion injury risk.

## 2. Materials and methods

### 2.1. Participants

**Fig 1:**
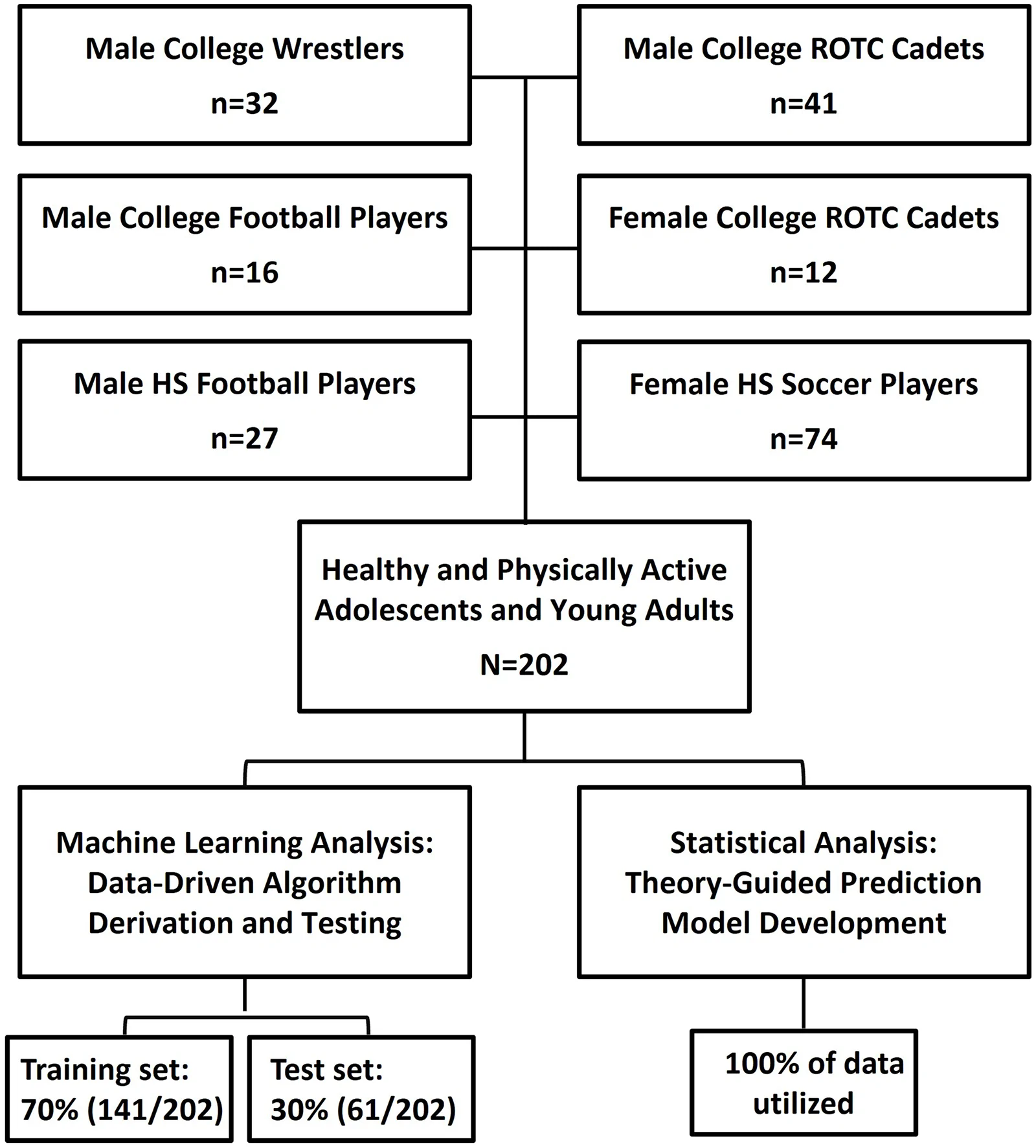
Data aggregation from different cohorts and retrospective cross-sectional analyses.

A retrospective and cross-sectional study design aggregated immersive VR test results and survey responses pertaining to concussion history for 202 physically active adolescents and young adults who had previously participated in other research projects between January 20, 2022 and June 13, 2023 (116 males: age 19,6 ± 3.1 years, height 178.7 ± 7.8 cm, mass 83.4 ± 17.8 kg; 86 females: age 15.7 ± 2.4 years, height 164.2 ± 6.0 cm, mass 57.9 ± 7.6 kg) [51–54]. The only exclusionary criterion for the prior studies was an injury-related impairment that limited the ability to perform rapid arm reaching and lower extremity lunging movements. The Institutional Review Board of the University of Tennessee at Chattanooga approved the study procedures (16–122, 22–071, 23–052), which included electronic documentation of informed consent and permission to access sport-related injury records for the research purpose from adult participants, as well as parents or guardians of minor participants, and the assent of minor participants. All archived data files were anonymized prior to their aggregation on January 11, 2024, which eliminated all personally identifiable information. A flow diagram depicts the membership of six cohorts that had previously performed the VR test, and the procedures used to analyze the data (Fig 1).

### 2.2. Procedures

**Fig 2:**
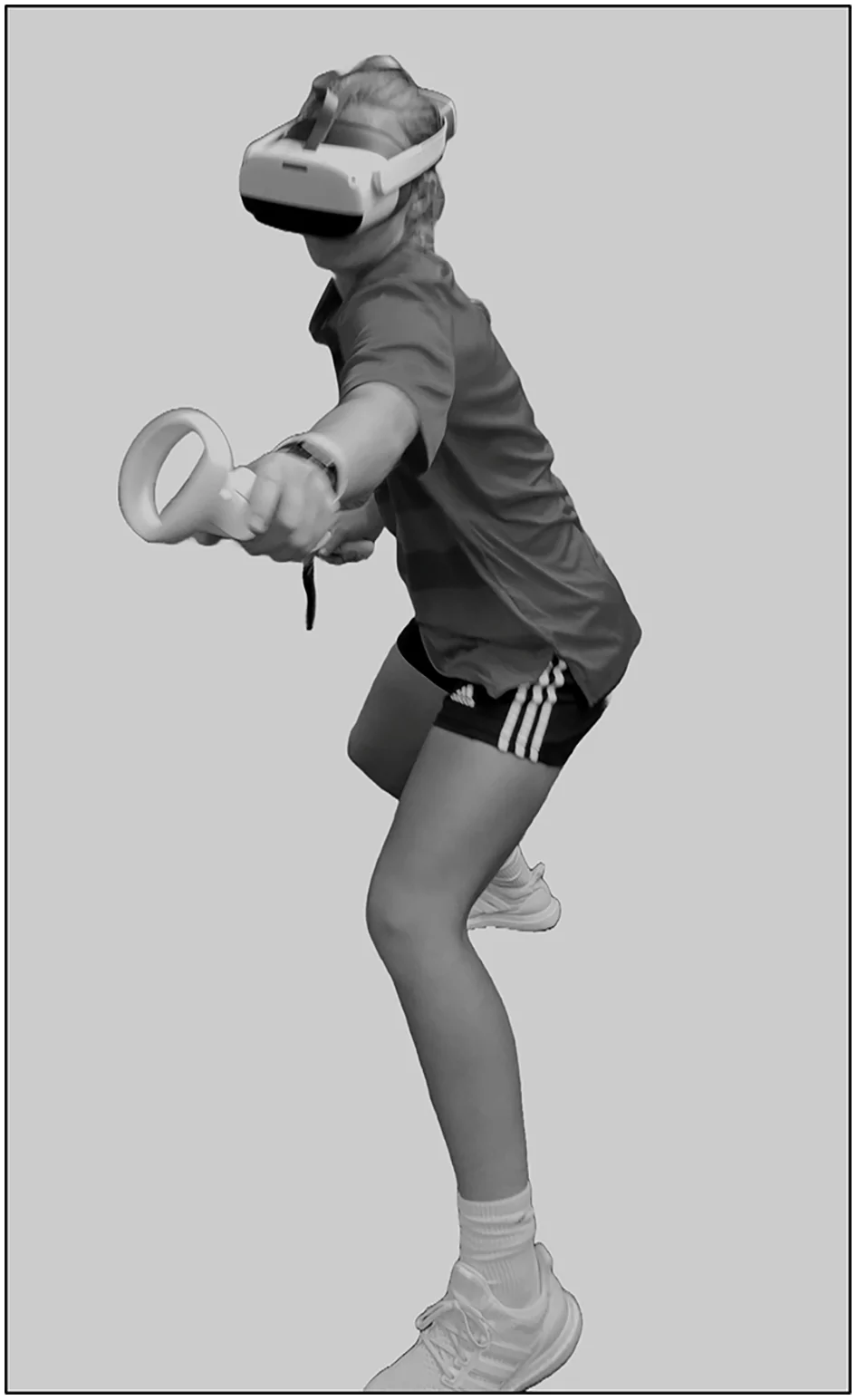
Immersive VR test movement response to a visual stimulus moving horizontally across the headset display. Simultaneous neck rotation, arm reaching, and single step lunging toward virtual response target in right or left direction (Reproduced with permission [54]).

The immersive VR system (PICO Neo3 Pro Eye, PICO Immersive, Ltd., Mountain View, CA, USA) presented a two-alternative forced-choice task that was initiated by the appearance of either a circle (i.e., filled interior) or ring (i.e., unfilled interior) that moved horizontally across the headset display. The stimuli initially appeared in either the center or a far peripheral location of the display and moved in either a leftward or rightward direction. A correct response to a filled circle’s movement direction was executed in the same direction (e.g., rightward circle movement paired with a congruent right response direction), whereas a correct response to an open ring’s movement direction was executed in the opposite direction (e.g., rightward ring movement paired with a left incongruent response direction). The movement response combined neck rotation, arm reaching, and single step lunging (Fig 2), which was required to make hand controller contact with a virtual response target (i.e., a green spherical object located beyond the individual’s maximum arm reach distance and outside the visual field without neck rotation).

Participant familiarity with the test was limited to a brief tutorial and four guided practice trials (i.e., visual and auditory instructions provided within the VR headset). A total of 40 successive trials were administered, with interstimulus intervals of 1.0, 1.5, or 2.0 seconds after the appearance of a central eye fixation cross that preceded each trial. Perceptual latency (PL) was defined as the time elapsed from stimulus appearance to a pre-defined velocity threshold (i.e., 15 degrees per second for neck rotation, 9 cm per second for arm reaching, and 5 degrees per second for single-step lunging), whereas response time (RT) was defined as the time elapsed from stimulus appearance to the maximum extent of movement toward the virtual response target. Failure to execute a valid response was assigned a default value of 1.25 seconds for PL and 2.5 seconds for RT.

A total of 13 VR performance metrics included 40-trial mean values for PL and RT of neck and arm movements and response accuracy. A rate correct per second (RCS) metric was calculated to represent both the speed and accuracy of responses (i.e., number correct / sum of elapsed times for 40 trials), both in terms of PL (RCS-PL) and RT (RCS-RT) for neck and arm movements that were measured by sensors in the headset and hand controllers. No metrics for step movements were analyzed, because they were indirectly estimated from the headset and hand controller sensor displacements. Intra-individual PL variability (PLV) and RT variability (RTV) were defined by the standard deviation of all valid trials, which were calculated separately for each participant’s neck, arm, and step movements. Intraclass correlation coefficients representing the test-retest reliability of RCS-PL and RCS-RT for the neck and arm have been documented to range from 0.851 to 0.887, and a range of test-retest reliability values for trial-to-trial PLV and RTV for the neck and arm from 0.693 to 0.836 [55]. Other VR metrics included left-right asymmetries for PL and RT mean values for the neck, arm, and step responses, and conflict effect (i.e., incongruent – congruent mean values) for PL and RT of the neck and arm responses.

### 2.3 Data analysis

Supervised machine learning was used as an exploratory approach to identify VR metrics that provide accurate classification of concussion history among individuals with a no concussion (NC) history, single concussion (SC) history, or multiple concussion (MC) history. Binary status classifications were created for comparison of NC with ≥1 concussion (SC + MC), as well as comparison of ≤1 concussion (NC + SC) with MC. Features that demonstrated high bivariate correlations with others (Pearson r > 0.90) were excluded from a stepwise logistic regression procedure with 5-fold cross-validation. LASSO logistic regression (L1 penalty + balanced class weights) was selected as the primary model to address severe class imbalance without synthetic oversampling, which has been recommended for small-sample medical classification tasks [49,50].

Receiver operating characteristic (ROC) area under curve (AUC), Brier score (calibration), precision, recall, and F1-score were used to evaluate model performance for repeated, stratified 5-fold cross-validation (3 repeats = 15 folds total) on the training partition (70% of data, stratified split) and an independent hold-out test set (30%). Sensitivity to modeling choices was assessed by comparing class-weighted logistic regression, synthetic minority over-sampling technique (SMOTE) + logistic regression, XGBoost with scale_pos_weight, and LASSO logistic regression. The machine learning code utilized Python 3 (Python Software Foundation, Wilmington, DE), the implementation environment was Jupyter notebook (Anaconda, Inc., Austin, TX), and Scikit-learn library was used for model implementation (Version 1.6.0; Scikit-learn Developers, Paris, France). All theory-based statistical analyses utilized SPSS (Version 29.0; IBM Corporation, Armonk, NY).

Backward stepwise logistic regression, bivariate correlation, ROC, and cross-tabulation analyses were performed on the full dataset to assess the consistency of the machine learning output with theorized relationships to components of the drift-diffusion model of decision-making. No alpha-level adjustment for multiple comparisons was used for hypothesis testing [56], but *p*-values associated with test results are reported. Because the purpose of the study was exploratory, and participants were not randomly selected, effect magnitudes (r^2^, AUC, odds ratio [OR]) and the OR 95% confidence interval (CI) were used as criteria for interpretation of the plausibility and meaningfulness of the results. Specifically, Analysis of Credibility was used to assess OR values with a CI lower limit >1.0 as statistically significant and meaningful estimates of effect magnitude in relation to a Skepticism Limit (SL) value [57]. Potential confounding factors were assessed with Tarone’s test for homogeneity of ORs across strata, and a weighted average of stratum-specific ORs was derived from the Mantel-Haenszel estimation procedure (OR_MH_).

## 3. Results

The dataset was highly imbalanced in terms of self-reported concussion history, which comprised 68% (138/202) NC, 20% (40/202) SC, and 12% (24/202) MC. Neither the machine learning nor the statistical analyses identified any factor that substantially differentiated NC from SC + MC (i.e., 0 versus ≥1 concussion). The iterative machine learning procedures identified 4 features that consistently discriminated NC + SC from MC (i.e., ≤1 concussion versus 2 or more concussions), which included Neck RCS-PL, Arm RCS-PL, Arm RCS-RT, and Sex (Male). Application of the LASSO model to the 61 cases reserved as a test set yielded AUC = 0.733, 86% sensitivity (recall), 67% specificity, 25% precision, and a Brier score of 0.234 (0.5 threshold). Comparisons of results derived from the four machine learning approaches are presented in supplementary tables.

Cross-tabulation analysis of the entire dataset failed to reveal a significant difference between sexes for the prevalence of MC, which was 14% (16/116) for males and 9% (8/86) for females, χ^2^(1)=0.95, *p* = 0.384. Separate logistic regression analyses for prediction of MC consistently demonstrated greater Exp(B) adjusted odds values for Males compared to Females (OR_Adj_), but Sex was not retained as a model covariate with any of the 4 VR metrics included in the machine learning model: Neck RCS-PL with Sex OR_Adj_ = 1.62, *p* = 0.306; Arm RCS-PL with Sex OR_Adj_ = 1.56, *p* = 0.340; Arm RCS-RT with Sex OR_Adj_ = 1.58, *p* = 0.333; and Arm RTV with Sex OR_Adj_ = 1.43, *p* = 0.445. There was also a lack of a statistically significant contribution of Age Category (i.e., College and High School) to the prediction models, despite College participants consistently demonstrating greater OR_Adj_ than High School participants for MC: Neck RCS-PL Age Category OR_Adj_ = 1.27, *p* = 0.595; Arm RCS-PL Age Category OR_Adj_ = 1.11, *p* = 0.813; Arm RCS-RT Age Category OR_Adj_ = 1.04, *p* = 0.933; and Arm RTV Age Category OR_Adj_ = 1.06, *p* = 0.890.

Consistent with the machine learning output for prediction of MC, synthesis of ongoing research involving female high school and college soccer players suggests Neck RCS-PL, Arm RCS-PL, or Arm RCS-RT as the best VR metrics for prospective discrimination of players who sustain a core or lower extremity sprain or strain from those who avoid such an injury. Furthermore, a meaningful bivariate correlation between RCS-PL and PLV has been consistently demonstrated, and both metrics have demonstrated dramatic post-training improvements [48]. Statistical analysis of the entire dataset of 178 NC + SC cases and 24 MC cases resulted in Neck RCS-PL AUC = 0.695, Arm RCS-PL AUC = 0.673, Arm RCS-RT AUC = 0.688, and Neck PLV AUC = 0.685. The AUC value for Neck RCS-PL alone closely corresponded to that for the 4-feature machine learning model (0.695 and 0.706, respectively).

Binary categorizations based on ROC cut-points demonstrated Neck RCS-PL ≥ 1.28 to discriminate MC from NC + SC, with χ^2^(1)=18.01, *p* < 0.001; 83% sensitivity, 62% specificity; OR=8.28 (CI: 2.72, 25.27); and SL = 2.02. Tarone’s test for homogeneity of ORs across the 5 cohorts, which differed in terms of Sex, Age Category, and Activity Category, did not identify any significant difference among them, with χ^2^(4)=5.81, *p* = 0.213; OR_MH_ = 7.00 (CI: 2.32, 21.17); SL = 2.14. Neck PLV ≤ 0.32 also provided good discrimination, with χ^2^(1)=11.71, p < 0.001; 75% sensitivity, 62% specificity; OR=4.85 (CI: 1.84, 12.83); and SL = 2.13. Tarone’s test for did not identify any significant difference in ORs among the 5 cohorts, with χ^2^(4)=6.27, *p* = 0.180; OR_MH_ = 4.22 (CI: 1.62, 11.01); SL = 2.35.

**Fig 3:**
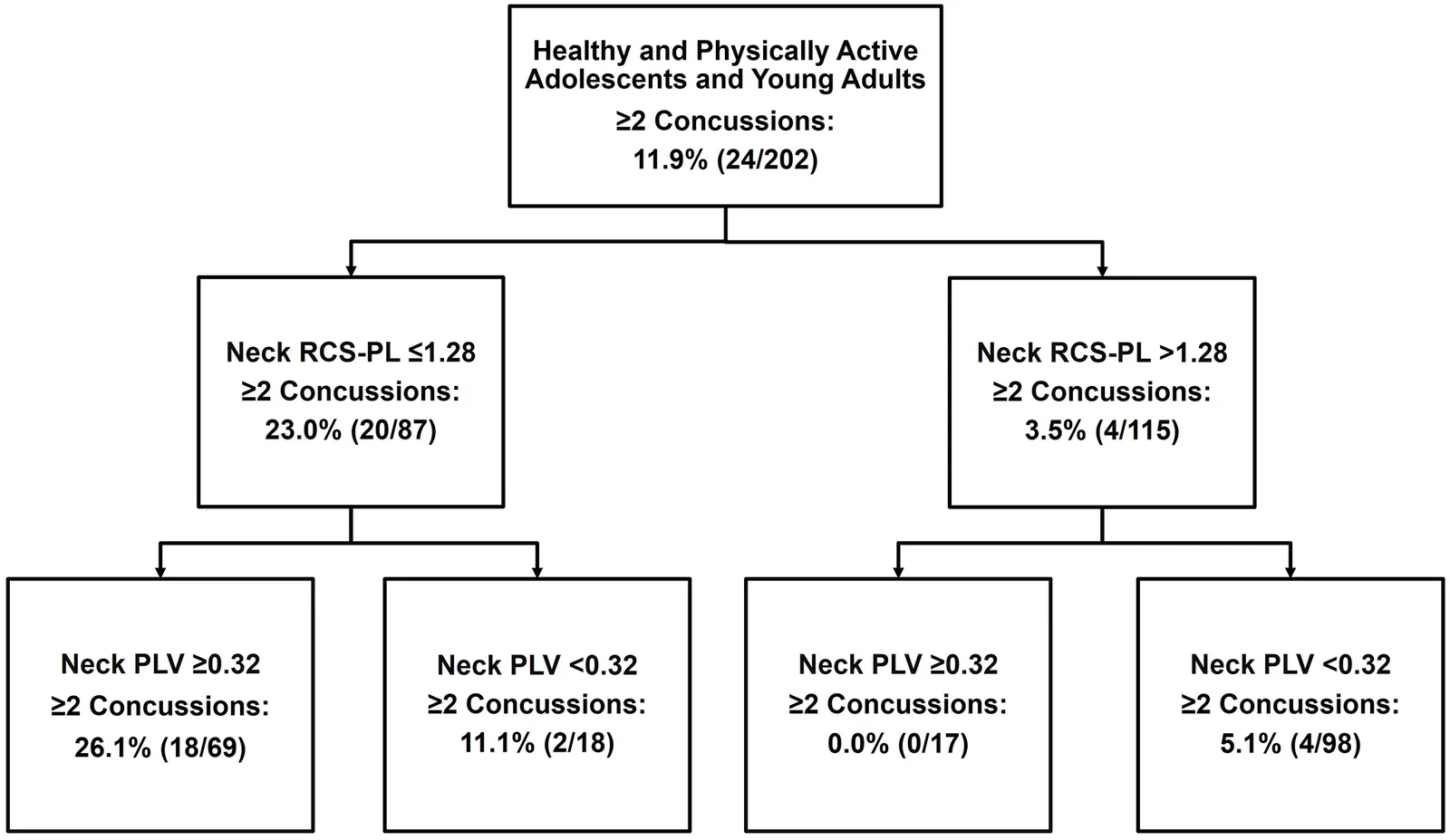
Prevalence of multiple concussion cases (≥2) for binary classifications of strongest discriminating factors. VR metrics: Neck Rate Correct per Second – Perceptual Latency (RCS-PL) and Neck Perceptual Latency Variability (PLV).

**Fig 4:**
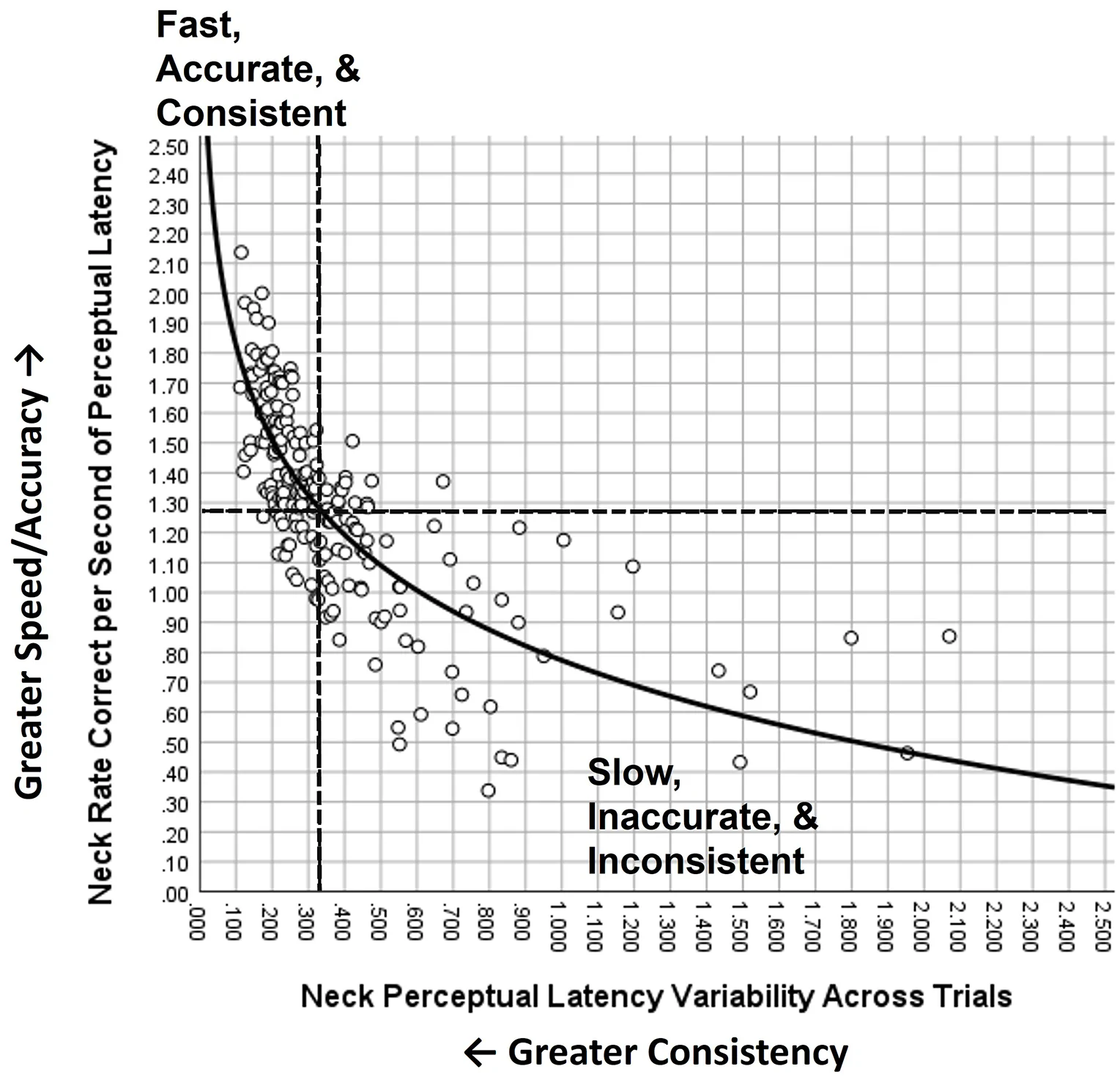
Inverse logarithmic correlation between metrics representing speed-accuracy tradeoff and across-trials inconsistency. VR metrics: Neck Rate Correct per Second – Perceptual Latency (RCS-PL) and Neck Perceptual Latency Variability (PLV).

The combination of Neck RCS-PL and Neck PLV may have clinical utility for identification of cumulative concussion effects (Fig 3). A meaningful inverse logarithmic correlation between Neck RCS-PL and Neck PLV was observed (Pearson r=−0.796, p < 0.001; Spearman’s ρ=−0.807, p < 0.001), which may be highly relevant to perceptual decision-making and initiation of an appropriate motor action (Fig 4). Derivation of an “Action Initiation Efficiency” (AIE) index by dividing Neck RCS-PL by Neck PLV discriminated MC from NC + SC with AUC = 0.691. A cut-point of AIE ≤ 3.54 demonstrated χ^2^(1)=14.73, *p* < 0.001; 75% sensitivity, 66% specificity; OR=5.75 (CI: 2.17, 15.25); and SL = 1.92. Tarone’s test did not identify any significant difference in ORs across the 5 cohorts, with χ^2^(4)=5.09, *p* = 0.278; OR_MH_ = 5.15 (CI: 1.92, 13.80); SL = 2.10.

## 4. Discussion

### 4.1 Interpretation of study findings

We used immersive VR to simulate sport demands for rapid processing of visual stimuli and decision-making, which are believed to be proportional to sport performance capabilities [35,58], as well as injury risk [30,59]. Decision-making involves somewhat distinct neuronal processes from those involved in sensorimotor control, but their interaction is critical for rapid execution of effective responses to rapidly changing environmental conditions [1,36,60]. Although RT (i.e., time elapsed from stimulus appearance to completion of a response) is a widely used behavioral measure of brain processing speed, PL (i.e., time elapsed from stimulus appearance to initiation of movement) may provide a better quantitative representation of the perceptual-cognitive component of responses. Furthermore, processing speed needs to be interpreted in relation to decision-making accuracy (i.e., correct direction) [32,35,38], which was represented by the RCS metric (i.e., number correct per unit of time). Consistent with our expectation derived from previous work with the same proprietary VR system [52–54,61], both the machine learning and theory-based statistical regression analyses identified Neck RCS-PL as the most influential VR metric for discrimination of MC cases from NC + SC cases. A potential bias derived from a disclosed conflict of interest and excessive reliance on prior work in formulation of expected findings makes future independent replication of this finding important.

Despite a lack of clear-cut distinctions between definitions of machine learning and statistical modeling [49], clinicians need to understand major differences in the respective processes that generate their results [50]. Supervised machine learning involves the input of “features” and the specification of an “outcome” to produce a “data-driven algorithm” that optimizes prediction accuracy, whereas a theory-based statistical method explicitly utilizes domain knowledge to specify a model for the direction and magnitude of the effect a “variable” is expected to demonstrate. A supervised machine learning algorithm may better identify complex interrelationships among predictive factors than a reductionist method designed to isolate a hypothesized effect [48], but a lack of explanation for the precise manner in which algorithm components contribute to the predicted outcome complicates interpretation of the output. Our machine learning analysis did incorporate domain knowledge to the extent that previous “feature engineering” (i.e., transformation or combination of variables) resulted in the inclusion of derived metrics known to associate with history of concussion (i.e., RCS-PL and RCS-RT for neck and arm movements), but the nature of interactions among the identified features is not clear. Thus, we used well-understood statistical methods for prediction modeling to assess the findings of the machine learning analysis in the context of domain knowledge [50].

Because our supervised machine learning method excluded potentially predictive metrics that were highly correlated with others, the model outputs did not include any measure of intra-individual variability across the 40 trials (i.e., PLV or RTV for neck and arm movements). Although we did not model trial-level correspondence of Neck RCS-PL to a “decision threshold” for initiation of a motor response, nor. Neck PLV to a “drift rate” of evidence accumulation, these quantifiable behavioral indicators can be conceptually viewed as averaged approximations of changing decision thresholds and drift rates across successive trials. The drift-diffusion computational model of decision-making has been described as a quantitative description of brain activations in perceptual-motor areas that correspond to behavioral outputs [38], which predicts that a relationship should exist between measures of speed-accuracy tradeoff (e.g., RCS-PL) and across-trial variability (e.g., PLV) [32]. Drift refers to the rate at which sensory inputs provide evidence that accumulates toward a decision threshold for executing a response in one of two directions, whereas diffusion refers to the adverse effects of intrinsic and extrinsic neural noise on signal processing [60,62]. The term drift-diffusion specifically relates to evidence accumulation in the area of the lateral intraparietal sulcus, whereas frontal and subcortical areas continuously monitor the speed and accuracy of successive response decisions and make adjustments that increase or decrease the decision threshold for subsequent responses [63]. Variation in the intrinsic functional connectivity of brain networks from moment to moment provides an explanation for variable behavioral responses to identical sensory stimuli [64]. Faster convergence to an optimal brain processing state is believed to result in less across-trial variability, as well as fast and accurate decisions [32]. On the basis of a theoretical expectation derived from the drift-diffusion model of decision-making, inclusion of Neck PLV in our statistical analyses of the entire dataset demonstrated an inverse natural logarithmic correlation with Neck RCS-PL (Fig 4), as well as a comparable level of predictive value for discrimination of MC cases from NC + SC cases (i.e., receiver operating characteristic AUC = 0.695 for Neck RCS-PL and AUC = 0.685 for Neck PLV).

Chronic neck dysfunction following concussion is common [65,66], and the drift-diffusion model of decision-making incorporates an inherent speed-accuracy tradeoff that reflects the threshold at which the accumulation of noisy sensory inputs triggers the initiation of a motor response [59]. Drift rates for post-acute concussion patients have been reported to be slower than those of control participants, with no difference in speed of motor output after a decision was made [60], which supports PL as a potentially better indicator of inefficient neural processing than RT. Microstructural disruption of white matter tracts [39], including focal axonal swellings [67], are believed to produce neural noise that increases variability in behavioral responses. Event-related desynchronization of alpha oscillations, which relates to a change in brain state that enhances the efficiency of communication between spatially separated brain areas, has been shown to be adversely affected by multiple concussions (i.e., ≥2) [8]. Collectively, the findings of these investigations are consistent with our interpretation of Neck RCS-PL and Neck PLV as potential behavioral indicators of a cumulative adverse effect of multiple concussions on the neural processes involved in making perceptual decisions.

### 4.2 Study limitations

An inherent limitation of concussion research is class imbalance, which is due to the relatively small number of concussion occurrences over the course of a given team’s sport season, and an even smaller number of athletes who have sustained more than one concussion over multiple years of sport participation. Among 3,278 American adolescent students, a history of a single concussion was reported by 18% and a history of 2 or more concussions was reported by only 7% [68]. Similar prevalence was documented for 15,343 American college athletes, with a history of a single concussion reported by 20% and history of 2 or more concussions reported by 7% [45].

A key limitation of our study is its retrospective and c\ross-sectional design, which lacked rigorous control of potential confounding factors that could be achieved through random selection of the participants. A large sex imbalance in the aggregated cohort precluded a definitive determination of its possible interaction effect with history of MC on perceptual decision-making efficiency. The machine learning results suggested male sex to be an important factor for accurate prediction, but its possible interaction with VR metrics is not clear [50], and our theory-based statistical analyses failed to identify a statistically significant male sex effect. Some evidence suggests that females may be more vulnerable to persistent effects from MC than males, and that sex differences in visual memory and visuospatial performance may be specific to the task used for testing [20]. Previous research has documented that suboptimal performance on our VR test is prospectively associated with sport-related injury in adolescent female soccer players [53,54], as well as college female soccer players [61]. Reliance on self-report of concussion history is widely recognized as an inherent limitation of most concussion-related research, due to lack of access to medical records that can confirm a history of diagnosed concussion(s) and the amount of time since the most recent occurrence [20]. If a history of SC or MC was not reported, the resulting misclassification may have resulted in an underestimate of associations with the immersive VR test metrics.

### 4.3 Future research

Both the supervised machine learning and theory-based statistical regression methods used in this study represent an exploratory approach involving both deductive and inductive reasoning to identify VR metrics that may have clinical value for the detection of persisting concussion effects and elevated risk for future injury. Arguably, a conventional hypothetico-deductive framework for determination of the statistical significance of differences between groups is not well-suited to advance understanding of the nature of complex brain-behavior relationships related to sport performance capabilities and injury avoidance, nor the identification of individuals whose behavioral metrics suggest a state of neural dysfunction. Despite the inherent limitations of an exploratory approach to a complex problem, it may represent an important initial step in building an evidence base for a new approach to concussion management. A lack of any meaningful differences between NC and ≥1 concussion (SC + MC), combined with the finding of substantial and inherently credible differences between ≤1 concussion (NC + SC) and MC, may be interpreted as evidence of a cumulative effect that is manifested after a second concussion. Such an interpretation is consistent with the findings of multiple studies that have documented greater adverse effects of MC compared to those of SC [5–8,10,11,13,14,25].

Currently, there is no optimal behavioral indicator of impaired perceptual-cognitive function that is feasible for clinical use [19], but intra-individual variability may be a marker of elevated post-concussion vulnerability of the brain to subsequent injury [40]. Training activities that incorporate decision-making appear to provide the potential for improvement of perceptual-cognitive efficiency [27,42]. Previous studies that utilized the same immersive VR test and similar VR training activities have demonstrated substantial improvements in performance speed, accuracy, and across-trial consistency [52,53], as well as lower injury incidence for trained athletes compared to non-trained athletes [53]. The findings of this exploratory study suggest that both Neck RCS-PL and Neck PLV have good potential for identification of individuals with suboptimal perceptual decision-making capabilities that might be modifiable through perceptual-cognitive training. The derived “Action Initiation Efficiency” metric (i.e., Neck RCS-PL divided by Neck PLV) may provide a useful behavioral approximation of the efficiency of neural processing involved in visual stimulus encoding, decision-making, and motor planning, but independent replication is needed to build confidence in the clinical utility of our findings.

## 5. Conclusions

Both supervised machine learning and theory-based statistical regression analyses identified the combined speed and accuracy of two-alternative forced-choice neck movements (i.e., Neck RCS-PL) as a potentially meaningful behavioral predictor of having previously sustained ≥2 concussions. The theory-based statistical analysis also yielded inherently credible findings that may approximate across-trial averages of the components of the well-validated drift-diffusion computational model of decision-making, with Neck RCS-PL relating to an averaged decision threshold and across-trial variability (i.e., Neck PLV) relating to the averaged adverse effect of neural noise on the rate of evidence accumulation for successive trials. Collectively, our findings support the importance of perceptual decision-making as a key factor that may link observable behavioral performance to brain processing efficiency.

## Supporting information

S1 Data Study data. (XLSX)

S2 Data Legends for study data abbreviations. (DOCX)
