# Supplementary material for: Virtual reality, machine learning, and statistical modeling reveal an association between multiple concussion history and impaired perceptual decision-making
Source: PLoS One. 2026 Sep 21;21(9):e0358042. doi: 10.1371/journal.pone.0358042 (PMC13592604; doi:10.1371/journal.pone.0358042)
Supplement: Supplementary file 1 [file pone.0358042.s002.docx]

| Variable | Abbreviation |
| --- | --- |
| History of Multiple Concussions (Coding: 0 or 1 = 0; ≥ 2 = 1) | HxSRC_0_1_vs_2plus |
| Sex (Male = 0; Female = 1) | Sex |
| Neck (Rotation) Perceptual Latency Rate Correct per Second | Neck_RCS_PL |
| Neck (Rotation) Perceptual Latency Variability (40-Trial SD) | Neck_PLV |
| Arm (Reach) Perceptual Latency Rate Correct per Second | Arm_RCS_PL |
| Arm (Reach) Response Time Rate Correct per Second | Arm_RCS_RT |
| Neck (Rotation) Response Time Rate Correct per Second | NeckRCS_RT |
| Arm (Reach) Perceptual Latency Variability (40-Trial SD) | Arm_PLV |
| Arm (Reach) Response Time Variability (40-Trial SD) | Arm_RTV |
| Neck (Rotation) Response Time Variability (40-Trial SD) | Neck_RTV |
| Step (Lunge) Perceptual Latency Variability (40-Trial SD) | Step_PLV |
| Step (Lunge) Response Time Variability (40-Trial SD) | Step_RTV |
| History of Concussion Number at Least 1 (Coding: 0 = 0; ≥ 1 = 1) | HxSRC |
| History of Concussion Total Number (Coding: 0 = 0; 1 = 1; ≥ 2 = 2) | HxSRC_0_1_2plus |
| History of Concussion Number Exactly 1 (Coding: 0 or ≥ 2 = 0; 1 = 1) | HxSRC_0_vs_1 |
| History of Concussion Number ≥ 2 (Coding: 0 = 0; ≥ 2 = 1) | HxSRC_0_vs_2plus |
| History of Concussion Number ≤ 1 vs. ≥ 2 (Coding: ≤ 1 = 0; ≥ 2 = 1) | HxSRC_1_vs_2plus |
| Number of Correct Responses (Congruent and Incongruent; 40Trials) | CorrectNumber |
| Percentage of Correct Responses (Congruent and Incongruent) | pcnt_correct |
| Percentage of Correct Responses (Congruent) | pcnt_correct_congruent |
| Percentage of Correct Responses (Incongruent) | pcnt_correct_incongruent |
