# Supplementary material for: Virtual reality, machine learning, and statistical modeling reveal an association between multiple concussion history and impaired perceptual decision-making
Source: PLoS One. 2026 Sep 21;21(9):e0358042. doi: 10.1371/journal.pone.0358042 (PMC13592604; doi:10.1371/journal.pone.0358042)
Supplement: Supplementary file 2 [file pone.0358042.s003.docx]

Dear authors,

Thank you for the submission of your manuscript, "Virtual reality, machine learning, and statistical modeling reveal an association between multiple concussion history and impaired perceptual decision-making." The topic is relevant, and the work has several innovative aspects. However, I believe there are substantial limitations that make it unsuitable for publication in its current form. Please find them listed below.

1. Retrospective, cross-sectional design, and self-reported concussion history.

Concussion history (0, 1, ≥2) is obtained retrospectively via self‑report, with no independent verification against medical or athletic records and no temporal information (e.g., time since last concussion, clustering of injuries). This introduces substantial misclassification risk and precludes any causal inference about cumulative concussion effects, which is a major concern.

2. Small and imbalanced multiple concussion group.

The key contrast (≤1 vs ≥2 concussions) relies on only 24 MC cases out of 202 participants, with strong class imbalance (68% NC, 20% SC, 12% MC). This severely limits statistical power, inflates uncertainty (e.g., very wide odds‑ratio confidence intervals), and makes ML models unstable, undermining the strength of the claimed “strong” discrimination.

3. Aggregation of six heterogeneous cohorts without rigorous control

Data are combined from six prior studies with different ages, sexes, and likely sports/exposure profiles, yet the heterogeneity across cohorts is not fully characterized or modeled (e.g., no cohort indicator in models, no stratified analyses). This raises serious concerns about selection bias and confounding that requires to be explicitly quantified and addressed.

4. Incomplete handling of confounding by sex, age, and sport/exposure

The sample is sex‑ and age‑imbalanced (older males, younger females), and MC prevalence may differ by sport and exposure, but sex and age are only superficially examined and inconsistently handled across ML and regression analyses. Failure to rigorously adjust for or explore these confounders weakens the internal validity of the claimed associations between VR metrics and concussion history.

5. Fragile machine learning pipeline and limited validation

The ML approach uses step-wise logistic regression with SMOTE and a single 70/30 train–test split, without nested cross‑validation, external validation, or calibration assessment. Given the small minority class, this is highly vulnerable to overfitting and optimistic performance estimates.

6. Use of SMOTE without adequate justification or sensitivity analysis

Synthetic minority over‑sampling (SMOTE) is applied to address imbalance, but there is no detailed justification, no exploration of alternative strategies (e.g., class weighting), and no systematic sensitivity analysis. Because SMOTE can distort the feature space and decision boundaries when the minority class is small and heterogeneous, this unexamined choice further reduces confidence in the reported discrimination.

7. Overstated mechanistic link to drift‑diffusion without formal modeling

The manuscript claims that Neck RCS‑PL and Neck PLV “approximate” the two components of a drift‑diffusion decision model, yet no formal sequential sampling model is fit to the trial‑level RT/accuracy distributions and no diffusion parameters (drift, boundary, non‑decision time, noise) are estimated.

8. Analytic flexibility and selective emphasis in VR metrics

A large set of VR metrics is computed (PL, RT, RCS‑PL, RCS‑RT for multiple segments, variability, asymmetries, conflict effects), but only a subset (mainly Neck RCS‑PL and Neck PLV) is emphasized, and the selection pathway is not fully transparent. Without a clear pre‑specified analysis plan and full reporting of all metrics considered, there is a risk of data‑driven selection that can inflate apparent effects.

9. Modest predictive performance with wide uncertainty

The main ML model for ≤1 vs ≥2 concussions achieve only moderate discrimination (AUC ~0.706), and the reported odds ratio for the test set (7.69) has an extremely wide 95% CI (0.74–79.48). Such imprecision and modest effect sizes, in the absence of external validation, limit the practical and scientific impact of the findings.

10. Conflict of interest and ecosystem dependence not fully integrated into interpretation

The lead author is a consultant for the company that developed the VR system used, and much of the rationale and support for the metrics relies on prior work with the same proprietary platform. While disclosed, this relationship is not deeply considered in the Discussion with respect to potential bias and the need for independent replication.

I believe that addressing these limitations would substantially improve the manuscript and make it suitable for publication.
