# Supplementary material for: Virtual reality, machine learning, and statistical modeling reveal an association between multiple concussion history and impaired perceptual decision-making
Source: PLoS One. 2026 Sep 21;21(9):e0358042. doi: 10.1371/journal.pone.0358042 (PMC13592604; doi:10.1371/journal.pone.0358042)
Supplement: Peer review history

# Peer review: Decision Letter 0

**DOI:** 10.1371/journal.pone.0358042.r001

**Authors:** Jose Tamez-Peña

19 Jan 2026

Dear Dr. Wilkerson,

Thank you for submitting your manuscript to PLOS ONE. After careful consideration, we feel that it has merit but does not fully meet PLOS ONE’s publication criteria as it currently stands. Therefore, we invite you to submit a revised version of the manuscript that addresses the points raised during the review process.

The manuscript is considered innovative but has fundamental methodological and analytical flaws that must be fixed before it can be considered for acceptance.

Core Problems Identified by the Reviewer:

Fatal Design Flaw: The study's retrospective, cross-sectional design using self-reported concussion history prevents any causal claims. The manuscript currently overstates its conclusions.

Severe Analytical Weaknesses:

Small, Imbalanced Sample: The key "multiple concussion" group is very small (n=24), undermining statistical power and machine learning (ML) stability.

Poorly Aggregated Data: Combining 6 different cohorts without properly accounting for their differences risks severe bias and confounding.

Inadequate ML Methods: The ML pipeline uses a weak validation method (single split) and applies a synthetic oversampling technique (SMOTE) without proper justification, making results prone to overfitting and unreliable.

Inconsistent Confounder Control: Critical variables like sex and age are not rigorously or consistently controlled for in the analyses.

Questionable Interpretation & Transparency:

Overstated Mechanism: Claims linking VR metrics to a theoretical decision-making model (drift-diffusion) are not supported by formal modeling.

"Cherry-Picking" Risk: Many VR metrics were analyzed, but the pathway to highlighting only a few is not transparent, raising concerns of selective reporting.

Overstated Results: The actual predictive performance is modest (AUC ~0.71) and highly uncertain (extremely wide confidence intervals), which is not adequately reflected in the narrative.

Please submit your revised manuscript by Mar 05 2026 11:59PM. If you will need more time than this to complete your revisions, please reply to this message or contact the journal office at plosone@plos.org. When you're ready to submit your revision, log on to https://www.editorialmanager.com/pone/ and select the 'Submissions Needing Revision' folder to locate your manuscript file.

- A letter that responds to each point raised by the academic editor and reviewer(s). You should upload this letter as a separate file labeled 'Response to Reviewers'.
- A marked-up copy of your manuscript that highlights changes made to the original version. You should upload this as a separate file labeled 'Revised Manuscript with Track Changes'.
- An unmarked version of your revised paper without tracked changes. You should upload this as a separate file labeled 'Manuscript'.

If you would like to make changes to your financial disclosure, please include your updated statement in your cover letter. Guidelines for resubmitting your figure files are available below the reviewer comments at the end of this letter.

If applicable, we recommend that you deposit your laboratory protocols in protocols.io to enhance the reproducibility of your results. Protocols.io assigns your protocol its own identifier (DOI) so that it can be cited independently in the future. For instructions see: https://journals.plos.org/plosone/s/submission-guidelines#loc-laboratory-protocols. Additionally, PLOS ONE offers an option for publishing peer-reviewed Lab Protocol articles, which describe protocols hosted on protocols.io. Read more information on sharing protocols at https://plos.org/protocols?utm_medium=editorial-email&utm_source=authorletters&utm_campaign=protocols.

We look forward to receiving your revised manuscript.

Kind regards,

Jose Gerardo Tamez-Peña, PhD

Academic Editor

PLOS One

Journal Requirements:

When submitting your revision, we need you to address these additional requirements.

1. Please ensure that your manuscript meets PLOS ONE's style requirements, including those for file naming. The PLOS ONE style templates can be found at

https://journals.plos.org/plosone/s/file?id=wjVg/PLOSOne_formatting_sample_main_body.pdf and

https://journals.plos.org/plosone/s/file?id=ba62/PLOSOne_formatting_sample_title_authors_affiliations.pdf

2. Please note that PLOS One has specific guidelines on code sharing for submissions in which author-generated code underpins the findings in the manuscript. In these cases, we expect all author-generated code to be made available without restrictions upon publication of the work. Please review our guidelines at https://journals.plos.org/plosone/s/materials-and-software-sharing#loc-sharing-code and ensure that your code is shared in a way that follows best practice and facilitates reproducibility and reuse.

3. Thank you for stating the following in the Competing Interests/Financial Disclosure section:

I have read the journal's policy and the authors of this manuscript have the following competing interests: Gary B. Wilkerson currently serves as a consultant to REACT Neuro (Cambridge, MA), which developed the immersive virtual reality system used for acquisition of the study data. The company had no role in the design of the study, analyses, interpretation of data, writing the manuscript, or the decision to publish the results. Neither of the other two authors declare any competing interests.

We note that one or more of the authors are employed by a commercial company: REACT Neuro (Cambridge, MA)

a. Please provide an amended Funding Statement declaring this commercial affiliation, as well as a statement regarding the Role of Funders in your study. If the funding organization did not play a role in the study design, data collection and analysis, decision to publish, or preparation of the manuscript and only provided financial support in the form of authors' salaries and/or research materials, please review your statements relating to the author contributions, and ensure you have specifically and accurately indicated the role(s) that these authors had in your study. You can update author roles in the Author Contributions section of the online submission form.

Please also include the following statement within your amended Funding Statement.

“The funder provided support in the form of salaries for authors [insert relevant initials], but did not have any additional role in the study design, data collection and analysis, decision to publish, or preparation of the manuscript. The specific roles of these authors are articulated in the ‘author contributions’ section.”

If your commercial affiliation did play a role in your study, please state and explain this role within your updated Funding Statement.

b. Please also provide an updated Competing Interests Statement declaring this commercial affiliation along with any other relevant declarations relating to employment, consultancy, patents, products in development, or marketed products, etc.

Within your Competing Interests Statement, please confirm that this commercial affiliation does not alter your adherence to all PLOS ONE policies on sharing data and materials by including the following statement: "This does not alter our adherence to PLOS ONE policies on sharing data and materials.” (as detailed online in our guide for authors http://journals.plos.org/plosone/s/competing-interests) . If this adherence statement is not accurate and there are restrictions on sharing of data and/or materials, please state these. Please note that we cannot proceed with consideration of your article until this information has been declared.

Please include both an updated Funding Statement and Competing Interests Statement in your cover letter. We will change the online submission form on your behalf.

4. We note that Figure 2 in your submission contain copyrighted images. All PLOS content is published under the Creative Commons Attribution License (CC BY 4.0), which means that the manuscript, images, and Supporting Information files will be freely available online, and any third party is permitted to access, download, copy, distribute, and use these materials in any way, even commercially, with proper attribution. For more information, see our copyright guidelines: http://journals.plos.org/plosone/s/licenses-and-copyright.

We require you to either (a) present written permission from the copyright holder to publish these figures specifically under the CC BY 4.0 license, or (b) remove the figures from your submission:

a. You may seek permission from the original copyright holder of Figure 2 to publish the content specifically under the CC BY 4.0 license.

We recommend that you contact the original copyright holder with the Content Permission Form (http://journals.plos.org/plosone/s/file?id=7c09/content-permission-form.pdf) and the following text:

“I request permission for the open-access journal PLOS ONE to publish XXX under the Creative Commons Attribution License (CCAL) CC BY 4.0 (http://creativecommons.org/licenses/by/4.0/). Please be aware that this license allows unrestricted use and distribution, even commercially, by third parties. Please reply and provide explicit written permission to publish XXX under a CC BY license and complete the attached form.”

Please upload the completed Content Permission Form or other proof of granted permissions as an "Other" file with your submission.

In the figure caption of the copyrighted figure, please include the following text: “Reprinted from [ref] under a CC BY license, with permission from [name of publisher], original copyright [original copyright year].”

b. If you are unable to obtain permission from the original copyright holder to publish these figures under the CC BY 4.0 license or if the copyright holder’s requirements are incompatible with the CC BY 4.0 license, please either i) remove the figure or ii) supply a replacement figure that complies with the CC BY 4.0 license. Please check copyright information on all replacement figures and update the figure caption with source information. If applicable, please specify in the figure caption text when a figure is similar but not identical to the original image and is therefore for illustrative purposes only.

5. We noted in your submission details that a portion of your manuscript may have been presented or published elsewhere. Please clarify whether this publication was peer-reviewed and formally published. If this work was previously peer-reviewed and published, in the cover letter please provide the reason that this work does not constitute dual publication and should be included in the current manuscript.

6. Please include captions for your Supporting Information files at the end of your manuscript, and update any in-text citations to match accordingly. Please see our Supporting Information guidelines for more information: http://journals.plos.org/plosone/s/supporting-information.

7. If the reviewer comments include a recommendation to cite specific previously published works, please review and evaluate these publications to determine whether they are relevant and should be cited. There is no requirement to cite these works unless the editor has indicated otherwise.

[Note: HTML markup is below. Please do not edit.]

Reviewers' comments:

Reviewer's Responses to Questions

**Comments to the Author**

1. Is the manuscript technically sound, and do the data support the conclusions?

Reviewer #1: Yes

Reviewer #2: Partly

**********

2. Has the statistical analysis been performed appropriately and rigorously?

Reviewer #1: Yes

Reviewer #2: No

**********

3. Have the authors made all data underlying the findings in their manuscript fully available?

The PLOS Data policy

Reviewer #1: Yes

Reviewer #2: Yes

**********

4. Is the manuscript presented in an intelligible fashion and written in standard English?

Reviewer #1: Yes

Reviewer #2: Yes

**********

Reviewer #1: .

Reviewer #2: Dear authors,

Thank you for the submission of your manuscript, "Virtual reality, machine learning, and statistical modeling reveal an association between multiple concussion history and impaired perceptual decision-making." The topic is relevant, and the work has several innovative aspects. However, I believe there are substantial limitations that make it unsuitable for publication in its current form. Please find them listed in the attached document.

**********

what does this mean?). If published, this will include your full peer review and any attached files.

If you choose “no”, your identity will remain anonymous but your review may still be made public.

**Do you want your identity to be public for this peer review?**  For information about this choice, including consent withdrawal, please see our Privacy Policy

Reviewer #1: No

Reviewer #2: No

**********

[NOTE: If reviewer comments were submitted as an attachment file, they will be attached to this email and accessible via the submission site. Please log into your account, locate the manuscript record, and check for the action link "View Attachments". If this link does not appear, there are no attachment files.]

To ensure your figures meet our technical requirements, please review our figure guidelines: https://journals.plos.org/plosone/s/figures

You may also use PLOS’s free figure tool, NAAS, to help you prepare publication quality figures: https://journals.plos.org/plosone/s/figures#loc-tools-for-figure-preparation.

NAAS will assess whether your figures meet our technical requirements by comparing each figure against our figure specifications.

Attachment Submitted filename: Reviews_1.docx

# Author comment: Author response to Decision Letter 1

**DOI:** 10.1371/journal.pone.0358042.r002

31 Mar 2026

We sincerely appreciate the opportunity to revise our manuscript, “Virtual reality, machine learning, and statistical modeling reveal an association between multiple concussion history and impaired perceptual decision-making," in a manner consistent with your supportive comments and recommendations. We believe the revisions have produced a substantially improved report. Our responses appear below each review comment.

Dear authors,

Thank you for the submission of your manuscript, "Virtual reality, machine learning, and statistical modeling reveal an association between multiple concussion history and impaired perceptual decision-making." The topic is relevant, and the work has several innovative aspects. However, I believe there are substantial limitations that make it unsuitable for publication in its current form. Please find them listed below.

1. Retrospective, cross-sectional design, and self-reported concussion history.

Concussion history (0, 1, ≥2) is obtained retrospectively via self‑report, with no independent verification against medical or athletic records and no temporal information (e.g., time since last concussion, clustering of injuries). This introduces substantial misclassification risk and precludes any causal inference about cumulative concussion effects, which is a major concern.

We understand the concern and fully acknowledge the limitations imposed by our retrospective, cross-sectional design, as well as reliance on self-report for identification of participants who had previously sustained one or more concussion events. Medical records are rarely accessible from all clinical sites where diagnostic or therapeutic services have been provided to the relatively small proportion of sport-related concussion cases that receive clinical medical care. Athletic records for concussion occurrences that preceded participation on a team at a given institution are almost exclusively reliant on self-report. Almost all concussion research requires the assumption that participants are truthfully responding to questions about their concussion history. Our previous work has not found time since most recent occurrence to be a meaningful factor for identification of individuals with persisting manifestations of a prior concussion. For those who have sustained more than one concussion, we have found participants to express a high degree of uncertainty in estimating the dates of the occurrences. Because false-negative responses (i.e., denial of past concussion occurrences) are much more likely than false-positive responses (i.e., report of events that did not occur), classification accuracy would likely increase, rather than decrease, the strength of the reported associations between multiple concussion history and VR perceptual-motor performance metrics. We do not want to imply that our findings support a cause-effect relationship. Impaired perceptual-motor function may have preceded concussion occurrence, or it could be a manifestation of persistent post-concussion dysfunction. In either case, the documented association is clearly worthy of consideration and further investigation. To acknowledge and address the noted limitations, the following content has been added to the Introduction section:

Numerous factors impose major limitations on research efforts to improve concussion diagnosis, prognosis, and long-term clinical management (Kenzie et al, 2017). Concussion has historically been associated with a discrete injury event, typically coinciding with a head impact, but delayed emergence of subtle and persistent abnormalities in brain function greatly complicates diagnostic accuracy (Masel & DeWitt, 2010). Almost all information available to clinicians for concussion diagnosis comes from patient-reported symptoms, which athletes are frequently reluctant to disclose (Katz et al, 2018), and existing clinical assessment metrics have questionable value for detection of subtle impairments (Chmielewski et al, 2021; Coffman et al, 2024). Even with a sufficiently sensitive clinical measure, the acquisition of clinical assessment data from enough concussed individuals to minimize the probability of a Type II hypothesis test error presents a formidable challenge (Bigler et al, 2013). Furthermore, a reductionist and hypothesis-driven approach is unlikely to identify complex, multivariable, and non-linear relationships that appear to be necessary for advancement of concussion management (Ahn et al, 2006).

2. Small and imbalanced multiple concussion group.

The key contrast (≤1 vs ≥2 concussions) relies on only 24 MC cases out of 202 participants, with strong class imbalance (68% NC, 20% SC, 12% MC). This severely limits statistical power, inflates uncertainty (e.g., very wide odds‑ratio confidence intervals), and makes ML models unstable, undermining the strength of the claimed “strong” discrimination.

The relatively low incidence of concussion (approximately 25-30% of high school and college athletes report a lifetime history of at least one concussion) makes a prospective study design impractical, even with an extremely large number of participants. Use of the term “strong” to qualitatively describe the discriminatory power of our prediction model has been changed to “meaningful” to avoid overstatement of the certainty of the findings. To acknowledge and address the noted limitations, the following content has been added to the Limitations subsection of the Discussion:

An inherent limitation of concussion research is class imbalance, which is due to the relatively small number of concussion occurrences over the course of a given team’s sport season, and an even smaller number of athletes who have sustained more than one concussion over multiple years of sport participation. Among 3,278 American adolescent students, a history of a single concussion was reported by 18% and a history of 2 or more concussions was reported by only 7% (Veliz et al, 2021). Similar prevalence was documented for 15,343 American college athletes, with a history of a single concussion reported by 20% and history of 2 or more concussions reported by 7% (Katz et al, 2018).

3. Aggregation of six heterogeneous cohorts without rigorous control

Data are combined from six prior studies with different ages, sexes, and likely sports/exposure profiles, yet the heterogeneity across cohorts is not fully characterized or modeled (e.g., no cohort indicator in models, no stratified analyses). This raises serious concerns about selection bias and confounding that requires to be explicitly quantified and addressed.

To address this concern, additional theory-based statistical analyses were conducted, and the following content has been added to the manuscript:

Potential confounding factors were assessed with Tarone’s test for homogeneity of ORs across strata, and a weighted average of stratum-specific ORs was derived from the Mantel-Haenszel estimation procedure (ORMH).

Neck RCS-PL ≥1.28: Tarone’s test for homogeneity of ORs across the 5 cohorts, which differed in terms of Sex, Age Category, and Activity Category, did not identify any significant difference among them, with χ2(4)=5.81, p=0.213; ORMH=7.00 (CI: 2.32, 21.17); SL=2.14.

Neck PLV ≤0.32: Tarone’s test for did not identify any significant difference in ORs among the 5 cohorts, with χ2(4)=6.27, p=0.180; ORMH=4.22 (CI: 1.62, 11.01); SL=2.35.

4. Incomplete handling of confounding by sex, age, and sport/exposure

The sample is sex‑ and age‑imbalanced (older males, younger females), and MC prevalence may differ by sport and exposure, but sex and age are only superficially examined and inconsistently handled across ML and regression analyses. Failure to rigorously adjust for or explore these confounders weakens the internal validity of the claimed associations between VR metrics and concussion history.

The response to the preceding comment described actions taken to address the possible impact of confounding factors for the theory-based statistical analysis. The following revised content pertains to the machine learning analysis, which has been added to the Data Analysis subsection of the manuscript:

Features that demonstrated high bivariate correlations with others (Pearson r >0.90) were excluded from a stepwise logistic regression procedure with 5-fold cross-validation. LASSO logistic regression (L1 penalty + balanced class weights) was selected as the primary model to address severe class imbalance without synthetic oversampling, which has been recommended for small-sample medical classification tasks (Christodoulou et al, 2019; Rodu et al, 2024). Receiver operating characteristic (ROC) area under curve (AUC), Brier score (calibration), precision, recall, and F1-score were used to evaluate model performance for repeated, stratified 5-fold cross-validation (3 repeats = 15 folds total) on the training partition (70% of data, stratified split) and an independent hold-out test set (30%). Sensitivity to modeling choices was assessed by comparing class-weighted logistic regression, synthetic minority over-sampling technique (SMOTE) + logistic regression, XGBoost with scale_pos_weight, and LASSO logistic regression.

5. Fragile machine learning pipeline and limited validation

The ML approach uses step-wise logistic regression with SMOTE and a single 70/30 train–test split, without nested cross‑validation, external validation, or calibration assessment. Given the small minority class, this is highly vulnerable to overfitting and optimistic performance estimates.

We thank the reviewer for highlighting these critical methodological issues. We agree that the original single-split approach with stepwise selection was vulnerable to overfitting in this small, imbalanced dataset (~12% multiple-concussion cases). In the revised manuscript we have implemented the following improvements, which have been added to the Data Analysis subsection and the Results section:

• Utilized a stratified 70/30 train-test split followed by repeated stratified 5-fold cross-validation (3 repeats = 15 folds total) on the training partition for robust performance estimation.

• Replaced SMOTE with LASSO logistic regression (L1 penalty + balanced class weights) as the primary model, since it yielded the highest test AUC values and strong minority-class recall in sensitivity analysis.

• Added the Brier score for explicit calibration assessment, which was not included in the original submission. Low values of Brier score are better and indicate higher reliability and accuracy.

• Final performance is reported on the completely independent hold-out test set (n=61, 7 positive cases). Test-set results for the primary LASSO model (threshold 0.5) are: AUC = 0.733, recall (class 1) = 0.857, precision (class 1) = 0.250, Brier score = 0.234. Cross-validation on training data yielded mean AUC = 0.597 ± 0.168 and mean recall = 0.511 ± 0.307 (high variability attributable to ~3–5 positives per validation fold). Full comparison across four modeling strategies is presented in Supplementary Table S1 (overall metrics) and Supplementary Table S2 (minority-class focus). Feature coefficients are shown in Supplementary Table S3. These changes substantially mitigate overfitting risk and provide more conservative, transparent, and calibrated estimates. The updated pipeline is described in the revised Methods section (2.3 Data Analysis).

6. Use of SMOTE without adequate justification or sensitivity analysis

Synthetic minority over‑sampling (SMOTE) is applied to address imbalance, but there is no detailed justification, no exploration of alternative strategies (e.g., class weighting), and no systematic sensitivity analysis. Because SMOTE can distort the feature space and decision boundaries when the minority class is small and heterogeneous, this unexamined choice further reduces confidence in the reported discrimination.

We sincerely thank the reviewer for this important critique. We agree that applying SMOTE without alternatives or sensitivity analysis was a limitation. In the revised analysis we have:

• Removed SMOTE as the default approach and adopted LASSO logistic regression (L1 penalty + balanced class weights) as the primary model — a more conservative and interpretable strategy that avoids synthetic oversampling and potential feature-space distortion.

• Conducted a direct sensitivity analysis comparing four strategies: (A) class-weighted logistic regression, (B) SMOTE + standard logistic regression, (C) XGBoost with scale_pos_weight, (D) LASSO logistic regression (L1 + class weights; primary). This provides a more robust assessment and helps minimize bias in our findings across the four modeling techniques (see Supplementary Tables S1–S3).

• Restricted predictors to the four most impactful features identified by stepwise selection with cross-validation.

• The LASSO model (D) yielded test AUC = 0.733, recall = 0.857, precision = 0.250, and Brier score = 0.234. Method B (SMOTE) produced a slightly lower AUC (0.730) with similar recall. Method C (XGBoost) showed poor minority-class detection (recall = 0.143). Method A (class-weighted logistic) was very close to D (AUC = 0.709, recall = 0.857).

We believe this revision fully addresses the reviewer's concern and enhances the credibility of the results.

7. Overstated mechanistic link to drift‑diffusion without formal modeling

The manuscript claims that Neck RCS‑PL and Neck PLV “approximate” the two components of a drift‑diffusion decision model, yet no formal sequential sampling model is fit to the trial‑level RT/accuracy distributions and no diffusion parameters (drift, boundary, non‑decision time, noise) are estimated.

No, we did not model trial-level correspondence of Neck RCS-PL to a “decision threshold” or. Neck PLV to the “drift rate” of evidence accumulation. We have added content to acknowledge this point, and to better describe the conceptual basis for linking the two VR metrics to the drift-diffusion model of perceptual decision-making. The following content has been added to the manuscript’s Discussion section:

Although we did not model trial-level correspondence of Neck RCS-PL to a “decision threshold” for initiation of a motor response, nor. Neck PLV to a “drift rate” of evidence accumulation, these quantifiable behavioral indicators can be conceptually viewed as averaged approximations of changing decision thresholds and drift rates across successive trials.

8. Analytic flexibility and selective emphasis in VR metrics

A large set of VR metrics is computed (PL, RT, RCS‑PL, RCS‑RT for multiple segments, variability, asymmetries, conflict effects), but only a subset (mainly Neck RCS‑PL and Neck PLV) is emphasized, and the selection pathway is not fully transparent. Without a clear pre‑specified analysis plan and full reporting of all metrics considered, there is a risk of data‑driven selection that can inflate apparent effects.

We thank the reviewer for this important comment on analytic flexibility and transparency in feature selection. We agree that the original submission did not sufficiently document the full set of computed VR metrics or clearly describe the selection pathway, which could raise concerns about potential data-driven bias. To reduce multicollinearity and redundancy, a correlation filter (Pearson r > 0.90) was applied as a pre-processing step. From the remaining features, stepwise logistic regression with 5-fold cross-validation and a class-weighted base model (scoring = 'roc_auc' to prioritize discrimination under imbalance) was used to identify the most influential predictors for discriminating NC+SC from MC. This process selected four features: Neck RCS-PL, Arm RCS-PL, Arm RCS-RT, and Sex. These features were the most impactful on the outcome as determined by the stepwise procedure. Stepwise selection with cross-validation is a valid and widely accepted exploratory approach in medical and sports science for high-dimensional behavioral data, particularly when the goal is to identify a parsimonious set of predictors from a large initial pool (Christodoulou et al., 2019; Rodu et al., 2024). The cross-validation scoring criterion and class weighting were pre-specified to mitigate overfitting and account for imbalance. The selection pathway is now fully docum

# Peer review: Decision Letter 1

**DOI:** 10.1371/journal.pone.0358042.r003

**Authors:** Jose Tamez-Peña

26 Aug 2026

Virtual reality, machine learning, and statistical modeling reveal an association between multiple concussion history and impaired perceptual decision-making

PONE-D-25-40759R1

Dear Dr. Wilkerson,

We’re pleased to inform you that your manuscript has been judged scientifically suitable for publication and will be formally accepted for publication once it meets all outstanding technical requirements.

Within one week, you’ll receive an e-mail detailing the required amendments. When these have been addressed, you’ll receive a formal acceptance letter and your manuscript will be scheduled for publication.

An invoice will be generated when your article is formally accepted. Please note, if your institution has a publishing partnership with PLOS and your article meets the relevant criteria, all or part of your publication costs will be covered. Please make sure your user information is up-to-date by logging into Editorial Manager at Editorial Manager® and clicking the ‘Update My Information' link at the top of the page. For questions related to billing, please contact billing support.

If your institution or institutions have a press office, please notify them about your upcoming paper to help maximize its impact. If they’ll be preparing press materials, please inform our press team as soon as possible -- no later than 48 hours after receiving the formal acceptance. Your manuscript will remain under strict press embargo until 2 pm Eastern Time on the date of publication. For more information, please contact onepress@plos.org.

Kind regards,

Jose Gerardo Tamez-Peña, PhD

Academic Editor

PLOS One

Additional Editor Comments (optional):

Reviewers' comments:

Reviewer's Responses to Questions

**Comments to the Author**

Reviewer #2: All comments have been addressed

**********

2. Is the manuscript technically sound, and do the data support the conclusions?

Reviewer #2: Yes

**********

3. Has the statistical analysis been performed appropriately and rigorously?

Reviewer #2: Yes

**********

4. Have the authors made all data underlying the findings in their manuscript fully available?

The PLOS Data policy

Reviewer #2: No

**********

5. Is the manuscript presented in an intelligible fashion and written in standard English?

Reviewer #2: Yes

**********

Reviewer #2: Dear authors,

Thank you for your response. I believe the manuscript has improved substantially after addressing all my concerns. I believe it is now suitable for publication.

**********

what does this mean?). If published, this will include your full peer review and any attached files.

If you choose “no”, your identity will remain anonymous but your review may still be made public.

**Do you want your identity to be public for this peer review?**  For information about this choice, including consent withdrawal, please see our Privacy Policy

Reviewer #2: No

**********

# Editor report: Acceptance letter

**DOI:** 10.1371/journal.pone.0358042.r004

**Authors:** Jose Tamez-Peña

PONE-D-25-40759R1

PLOS One

Dear Dr. Wilkerson,

I'm pleased to inform you that your manuscript has been deemed suitable for publication in PLOS One. Congratulations! Your manuscript is now being handed over to our production team.

At this stage, our production department will prepare your paper for publication. This includes ensuring the following:

* All references, tables, and figures are properly cited

* All relevant supporting information is included in the manuscript submission,

* There are no issues that prevent the paper from being properly typeset

You will receive further instructions from the production team, including instructions on how to review your proof when it is ready. Please keep in mind that we are working through a large volume of accepted articles, so please give us a few days to review your paper and let you know the next and final steps.

Lastly, if your institution or institutions have a press office, please let them know about your upcoming paper now to help maximize its impact. If they'll be preparing press materials, please inform our press team within the next 48 hours. Your manuscript will remain under strict press embargo until 2 pm Eastern Time on the date of publication. For more information, please contact onepress@plos.org.

You will receive an invoice from PLOS for your publication fee after your manuscript has reached the completed accept phase. If you receive an email requesting payment before acceptance or for any other service, this may be a phishing scheme. Learn how to identify phishing emails and protect your accounts at https://explore.plos.org/phishing.

If we can help with anything else, please email us at customercare@plos.org.

Thank you for submitting your work to PLOS One and supporting open access.

Kind regards,

PLOS One Editorial Office Staff

on behalf of

Dr. Jose Gerardo Tamez-Peña

Academic Editor

PLOS One
